# Inhibition of PDE5 Restores Depressed Baroreflex Sensitivity in Renovascular Hypertensive Rats

**DOI:** 10.3389/fphys.2016.00015

**Published:** 2016-01-28

**Authors:** Clênia de Oliveira Cavalcanti, Rafael R. Alves, Alessandro L. de Oliveira, Josiane de Campos Cruz, Maria do Socorro de França-Silva, Valdir de Andrade Braga, Camille de Moura Balarini

**Affiliations:** ^1^Centro de Biotecnologia, Universidade Federal da ParaíbaJoao Pessoa, Brazil; ^2^Centro de Ciências Médicas, Universidade Federal da ParaíbaJoao Pessoa, Brazil; ^3^Centro de Ciências da Saúde, Universidade Federal da ParaíbaJoao Pessoa, Brazil

**Keywords:** resistant hypertension, sildenafil, baroreflex, nitric oxide, angiotensin-II

## Abstract

Renal artery stenosis is frequently associated with resistant hypertension, which is defined as failure to normalize blood pressure (BP) even when combined drugs are used. Inhibition of PDE5 by sildenafil has been shown to increase endothelial function and decrease blood pressure in experimental models. However, no available study evaluated the baroreflex sensitivity nor autonomic balance in renovascular hypertensive rats treated with sildenafil. In a translational medicine perspective, our hypothesis is that sildenafil could improve autonomic imbalance and baroreflex sensitivity, contributing to lower blood pressure. Renovascular hypertensive 2-kidney-1-clip (2K1C) and sham rats were treated with sildenafil (45 mg/Kg/day) during 7 days. At the end of treatment, BP and heart rate (HR) were recorded in conscious rats after a 24-h-recovery period. Spontaneous and drug-induced baroreflex sensitivity and autonomic tone were evaluated; in addition, lipid peroxidation was measured in plasma samples. Treatment was efficient in increasing both spontaneous and induced baroreflex sensitivity in treated hypertensive animals. Inhibition of PDE5 was also capable of ameliorating autonomic imbalance in 2K1C rats and decreasing systemic oxidative stress. Taken together, these beneficial effects resulted in significant reductions in BP without affecting HR. We suggest that sildenafil could be considered as a promising alternative to treat resistant hypertension.

## Introduction

Even though arterial hypertension is the most common modifiable risk factor for cardiovascular diseases, it is still an important health problem worldwide. It is predicted that its prevalence will reach 1/3 of global population by 2025 (Chokshi et al., 2013; Oparil and Schmieder, 2015). Stenosis of renal artery is frequently associated with resistant hypertension. In those patients, the usually available antihypertensive drugs protocol fail to normalize blood pressure (BP), even when in association (Faselis et al., 2011; Carey, 2013; Vongpatanasin, 2014). Therefore, the urgency in developing new approaches to treat arterial hypertension is clear. The experimental model of renovascular hypertension proposed by Goldblatt mimics human renovascular hypertension (Goldblatt et al., 1934; Navar et al., 1998). In that model, known as 2-kidney-1-clip (2K1C) model, the unilateral renal artery stenosis reduces renal perfusion and chronically stimulates the renin-angiotensin-aldosterone system (RAAS; Navar et al., 1998).

Several studies describe that the central control of BP is impaired during hypertension in humans and animal models, possibly due to autonomic dysfunction (Irigoyen and Krieger, 1998; Campagnaro et al., 2012; Grassi et al., 2015). Angiotensin II (Ang II) can directly affect central areas involved in autonomic control. The activation of type 1 Ang II receptor (AT1R) and the consequent production of reactive oxygen species (ROS) on different brain nuclei involved in autonomic control are currently well-accepted mechanisms for explaining autonomic dysfunction observed in arterial hypertension (Braga et al., 2011; de Queiroz et al., 2013). In recent studies, our group and others have shown that antioxidant therapy can reverse the reduced baroreflex sensitivity in spontaneously hypertensive rats (SHR) and in renovascular hypertensive rats (Nishi et al., 2010a; Botelho-Ono et al., 2011; Guimarães et al., 2012; Monteiro et al., 2012).

Sildenafil is a specific inhibitor of phosphodiesterase 5 (PDE5), the enzyme responsible for degrading cyclic guanosine monophosphate (cGMP). Thus, the main mechanism of action for this drug is the increase in nitric oxide (NO)/cGMP signaling pathway (Terrett et al., 1996; Palit and Eardley, 2010). In addition, our group has recently shown that sildenafil exhibits antioxidant properties (Balarini et al., 2013; Rodrigues et al., 2013; Dias et al., 2014b; Fahning et al., 2015; Leal et al., 2015). However, the efficacy of sildenafil in ameliorating central control of BP during arterial hypertension has not been investigated. In the present study, we aimed to evaluate the possible effects of sildenafil treatment on the central control of blood pressure through baroreflex sensitivity in 2K1C hypertensive rats. Furthermore, we evaluated whether the treatment was able to improve autonomic imbalance by reducing oxidative stress in those animals.

## Materials and methods

### Animals

Thirty eight Wistar rats (*Rattus norvegicus*) weighing 150–200 g were used. Animals were bred and housed in a temperature and humidity-controlled room set to a 12/12 h dark/light cycle with access to water and regular chow (Labina®, Purina, SP, Brazil) *ad libitum*. Experimental procedures were performed in accordance with National Institutes of Health (NIH) guidelines and protocols were approved by Institutional Animal Care and Use Committee (CEUA-UFPB protocol #042/2015).

### Study design

Animals were divided in four different groups: (1) Sham + vehicle (*n* = 9): animals were submitted to sham surgery and received vehicle during 7 days; (2) Sham + sildenafil (*n* = 7): animals were submitted to sham surgery and received sildenafil citrate during 7 days (45 mg/Kg/day) by gavage; (3) 2K1C + vehicle (*n* = 11): animals were submitted to renal artery clipping to induce renovascular hypertension and received vehicle during 7 days; and (4) 2K1C + sildenafil (*n* = 11): animals were submitted to renal artery clipping to induce renovascular hypertension and received sildenafil citrate during 7 days (45 mg/Kg/day by gavage). Sildenafil dose was chosen based on previous studies and on the difference on pharmacokinetics for sildenafil in different species (Walker et al., 1999; Ferreira-Melo et al., 2006; Guimarães et al., 2013). Baseline blood pressure (BP) and heart rate (HR), baroreflex function and autonomic tone evaluation test were performed in most of the animals for each group, including the ones used for typical raw tracings representation. Firstly, baseline BP and HR were recorded for 40 min. For drug-induced baroreflex function studies, phenylephrine and sodium nitroprusside were randomly administered with 15 min interval between doses in order to guarantee that cardiovascular parameters were back to baseline values. Lastly, propranolol and atropine were randomly administered with 3 h interval between doses. Details are given in specific protocols ahead. Body weight and cardiovascular baseline values for each experimental protocol is shown in Table 1 and Table 2, respectively.

**Table 1:** **Weight values from the different groups**.

	**Initial body weight (g)**	**Final body weight (g)**	**Right kidney/Body weight (mg/g)**	**Left kidney/Body weight (mg/g)**	**Heart weight/Body weight (mg/g)**	**Right kidney weight (g)**	**Left kidney weight (g)**	**Heart weight (g)**
Sham + Vehicle	173.8 ± 3.7	289.7 ± 13.8	4.4 ± 0.14	4.2 ± 0.17	3.7 ± 0.16	1.3 ± 0.04	1.2 ± 0.05	1.1 ± 0.07
Sham + Sildenafil	157.1 ± 1.2^a^	201.7 ± 12.6^a^	4.3 ± 0.20	4.3 ± 0.23	4.2 ± 0.28	0.85 ± 0.04^d^	0.9 ± 0.05^a^	0.83 ± 0.05^d^
2K1C + Vehicle	174.0 ± 2.8	272.8 ± 13.4	2.6 ± 0.37^b^, ^c^	4.3 ± 0.31	4.1 ± 0.22	0.74 ± 0.12^c^	1.1 ± 0.05	1.1 ± 0.05
2K1C + Sildenafil	175.8 ± 4.2	283.3 ± 7.6	2.5 ± 0.4^b^, ^c^	4.4 ± 0.31	3.6 ± 0.21	0.70 ± 0.13^c^	1.2 ± 0.08	1.0 ± 0.05

a *p < 0.01 vs. all other groups*.

b *p < 0.01 vs. both sham groups*.

c *p < 0.05 vs. left side from the same group*.

d *p < 0.05 vs. sham + vehicle*.

**Table 2:** **Values of MAP and HR before different drugs**.

	**Before baroreflex test**		**Before first autonomic blockade**		**Before second autonomic blockade**	
	**MAP (mmHg)**	**HR (bpm)**	**MAP (mmHg)**	**HR (bpm)**	**MAP (mmHg)**	**HR (bpm)**
Sham + Vehicle	116 ± 3	390 ± 12	124 ± 4	355 ± 10	126 ± 2	347 ± 10
Sham + Sildenafil	121 ± 7	370 ± 21	120 ± 6	369 ± 15	116 ± 6	383 ± 22
2K1C + Vehicle	177 ± 6	381 ± 13	186 ± 10	390 ± 15	176 ± 14	402 ± 21
2K1C + Sildenafil	144 ± 3	367 ± 9	151 ± 8	329 ± 10	149 ± 6	328 ± 18

*Values were compared by one-way ANOVA for the same group in the three different situations. No differences were found*.

### Renal artery clipping

The induction of renovascular hypertension was performed as previously standardized in our laboratory (Botelho-Ono et al., 2011; Queiroz et al., 2012). Briefly, animals were anesthetized with a mixture of ketamine and xylazine (75 and 10 mg/Kg, respectively, i.p.). The surgical procedures were executed only after the absence of withdraw and corneal reflexes. Right renal artery was carefully exposed through a retroperitoneal incision. A U-shaped silver clip (0.2 mm wide opening) was positioned around renal artery to decrease renal blood flow. Sham animals underwent the same surgical process, except for the implantation of the silver clip. After 5 weeks of surgery, the treatment protocol with sildenafil or vehicle was started and continued for 7 days.

### Hemodynamic measures

Six weeks after the induction of hypertension (or sham surgery), rats were anesthetized with a mixture of ketamine and xylazine (75 and 10 mg/Kg, respectively, i.p.) for catheters implantation and direct hemodynamic measurements. The surgical procedures were executed only after the absence of withdraw and corneal reflexes. Polyethylene catheters were inserted into the femoral artery and vein though a small inguinal incision in order to allowing blood pressure (BP) recordings and drug administration, respectively. Pulsatile arterial pressure was recorded using a pressure transducer (BRPL2, WPI, Sarasota, FL, USA) coupled to an amplifier and to an acquisition system (PowerLab, ADInstruments, Bella Vista, NSW, Australia) using a specific software (LabChart 5.0, ADInstruments, Bella Vista, NSW, Australia). Systolic arterial pressure (SAP), mean arterial pressure (MAP), diastolic arterial pressure (DAP), and heart rate (HR) were derived from the pulsatile arterial pressure online.

### Baroreflex sensitivity test

Baroreflex sensitivity was evaluated after a 24-h-recovery from the catheter implantation surgery. After 40 min of blood pressure and heart rate baseline recordings, reflex responses were obtained using vasoactive drugs as previously described using the modified Oxford method (Botelho-Ono et al., 2011; Guimarães et al., 2012; Alves et al., 2015). Briefly, a single bolus injection of phenylephrine (PHE, 8 μg/Kg) or sodium nitroprusside (SNP, 25 μg/Kg) was randomly given in order to elicit changes in blood pressure, which were similar in all groups. The second drug was administrated only after MAP and HR had returned to baseline values (15 min interval between drugs). Cardiac baroreceptor reflex responses were evaluated at the maximum (peak changes) responses (Giusti et al., 2011). Reflex changes in HR were quantified and plotted as changes in heart rate over changes in mean arterial pressure (ΔHR/ΔMAP, beats per minute/mmHg). Data were analyzed by linear regression using Prism 6 (GraphPad Software, Inc., San Diego, CA, USA) and the slope of linear regression provided baroreflex gain for each animal. Also, spontaneous baroreceptor reflex sensitivity (SBRS) was evaluated as previously described (Braga et al., 2008; Fazan et al., 2008) using the sequence method. A baroreflex sequence was defined as a sequence of at least four heart beats in which both systolic arterial pressure and pulse interval increased (up sequences) or decreased (down sequences). The gain of the baroreflex response was calculated and expressed as beats per minute/mmHg). Tracings were analyzed using HemoLab software (kindly provided by Dr. Harald Stauss, University of Iowa, version 9.3). The absolute values for DAP and SAP over which the spontaneous baroreflex was assessed were, respectively (in mmHg): sham + vehicle: 111 ± 12 and 141 ± 4; sham + sildenafil: 104 ± 7 and 143 ± 9; 2K1C + vehicle: 152 ± 6 and 209 ± 10; 2K1C + sildenafil: 117.5 ± 4 and 177 ± 4.

### Evaluation of autonomic control of heart rate

Autonomic function was assessed in conscious animals (Botelho-Ono et al., 2011; Xia et al., 2013). Autonomic blockade was performed to evaluate the contribution of sympathetic and parasympathetic nervous system to heart rate control. Changes in heart rate after the injection of propranolol (β-blocker, 5 mg/Kg, i.v.) or atropine (muscarinic receptor blocker, 4 mg/Kg, i.v.) were calculated and expressed as ΔHR. Variation in mean arterial pressure (ΔMAP) was also measured. Each drug administration was randomly chosen and separated from each other by an interval of at least 3 h on the same day. The first autonomic blockade was performed after MAP and HR had returned to baseline values from baroreflex studies (15 min). To assure that there was no interference of baroreflex drugs or the first autonomic blockage on autonomic responses, values for MAP and HR prior to baroreflex, prior to the first autonomic drug and prior to the second autonomic drug were provided in Table 2. No differences were found among treatments within the groups.

### Oxidative stress assay

After the blood pressure recording experiments, blood was collected through the venous catheter and centrifuged at 200 g, 4°C, during 15 min. Serum samples were collected for thiobarbituric acid reactive species (TBARS) assay. The concentration of malondialdehyde (MDA), an end product of lipid peroxidation, was measured as an indicative of oxidative stress. In this assay, MDA reacts with thiobarbituric acid to produce a red-colored complex. Briefly, 400 μL of perchloric acid (7%) was added to 250 μL serum, mixed and centrifuged at 600 g, 4°C, during 20 min. The supernatant was collected, added to 400 μL of thiobarbituric acid (0.6%), heat at 60°C during 1 h and read at 532 nm. A standard curve of MDA was constructed and the results were expressed as nmol of MDA/mL.

### Kidney and heart weight

After the experiments animals were euthanized with an overdose of thiopental (200 mg/Kg). Kidneys and heart were collected, cleaned from connective tissue and weighed.

### Statistical analyzes

All results are expressed as mean ± SEM. Statistical comparisons among groups were performed by *t*-test or one-way ANOVA followed by Tukey's *post hoc* when appropriate. Statistical analyzes were performed using Prism 6 and the differences were considered significant when *p* < 0.05.

## Results

### Body and organs weights

As shown in Table 1, animals from group sham + sildenafil were smaller than others. Considering that, organ weights were corrected by body weight. Clipped kidney from both renovascular hypertensive groups showed atrophy, as demonstrated by the reduction in right kidney weight/body weight ratio in relation to the other kidney in the same group. No differences were observed in left kidney weight/body weight ratio nor heart weight/body weight ratio regarding the induction of hypertension or treatment with sildenafil. Absolute values for organ weights were also provided in Table 1.

### Sildenafil treatment decreases blood pressure in hypertensive rats

**Figure 1:**
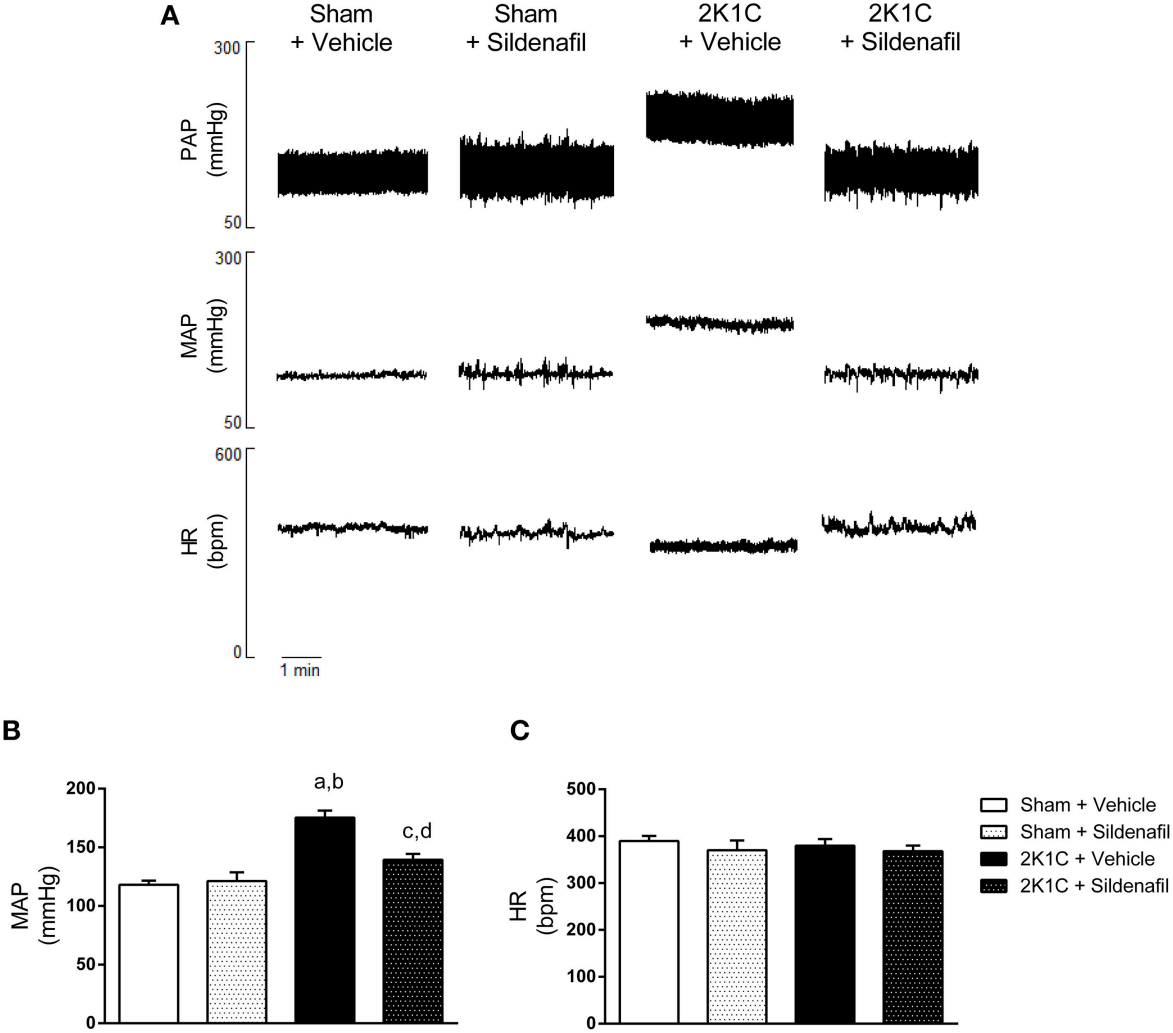
**Sildenafil reduces blood pressure in hypertensive rats**. **(A)** Representative tracings from one animal of each group (Sham + vehicle, Sham + sildenafil, 2K1C + vehicle, and 2K1C + sildenafil) showing pulsatile arterial pressure (PAP), mean arterial pressure (MAP), and heart rate (HR). **(B)** Effect of sildenafil treatment during 7 days on MAP (a, *p* < 0.01 vs. sham + vehicle; b, *p* < 0.01 vs. sham + sildenafil; c, *p* < 0.05 vs. sham + vehicle; d, *p* < 0.01 vs. 2K1C + vehicle). **(C)** Effect of sildenafil treatment during 7 days on HR.

The results of mean arterial pressure (MAP) and heart rate (HR) are summarized in Figure 1. Figure 1A shows representative tracings of pulse arterial pressure (PAP), MAP, and HR from one animal of each studied group. As expected, MAP was increased in 2K1C + vehicle animals when compared with sham + vehicle (175 ± 6 vs. 118 ± 3 mmHg, *p* < 0.01; Figure 1B). Sildenafil treatment reduced MAP when compared to non-treated hypertensive animals (139 ± 5 vs. 175 ± 6 mmHg, *p* < 0.01; Figure 1B). The administration of sildenafil to sham animals did not significantly modify MAP (121 ± 7 vs. 118 ± 3 mmHg). Conversely, HR was not different among all four groups (Figures 1A,C).

### Sildenafil treatment improves baroreflex sensitivity in hypertensive rats

**Figure 2:**
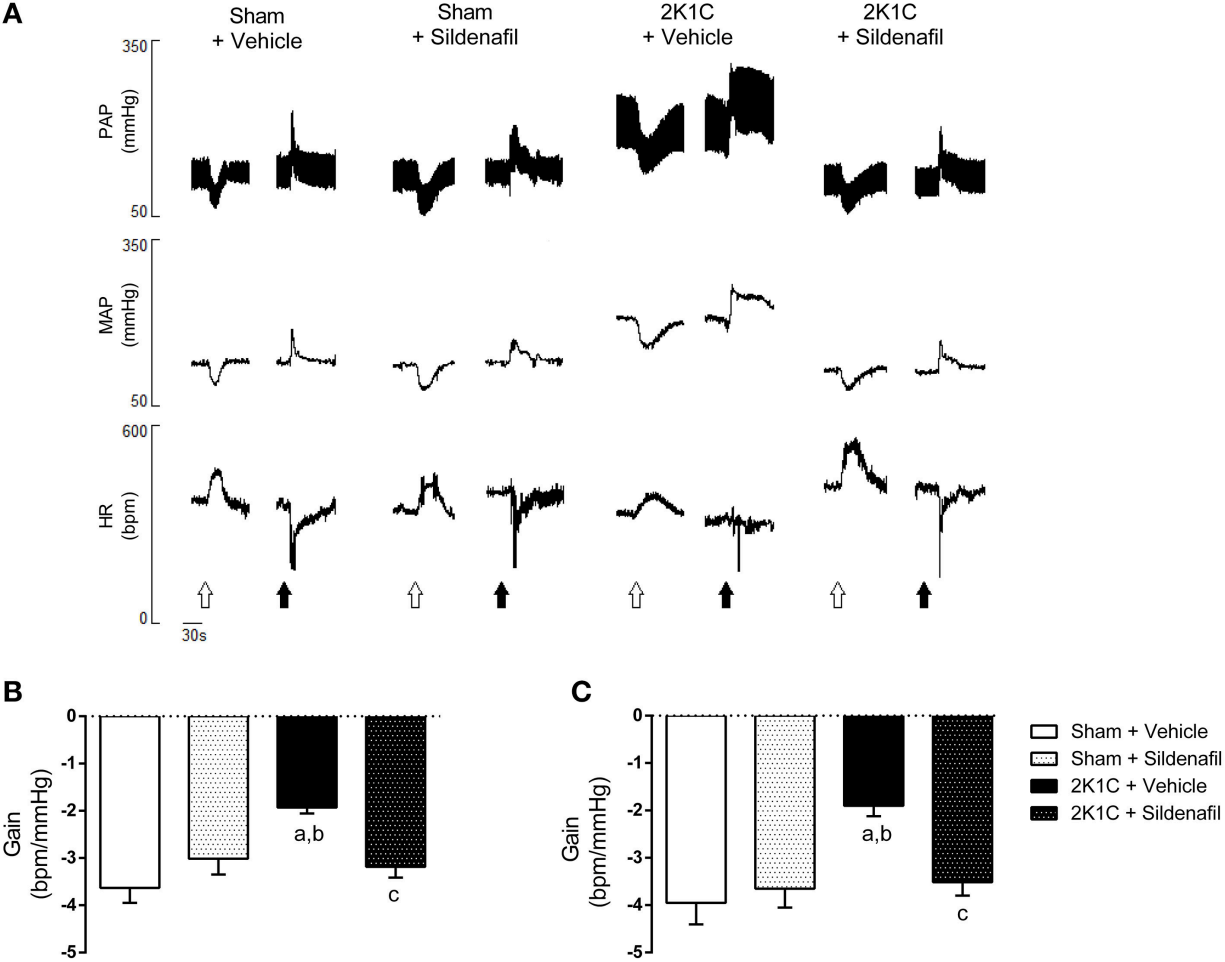
**Sildenafil restores baroreflex sensitivity in hypertensive rats**. **(A)** representative tracings from one animal of each group (Sham + vehicle, Sham + sildenafil, 2K1C + vehicle, and 2K1C + sildenafil) showing changes in pulsatile arterial pressure (PAP), mean arterial pressure (MAP), and heart rate (HR) in response to sodium nitroprusside (25 μg/Kg, open arrows) and phenylephrine (8 μg/Kg, black arrows). **(B)** effect of sildenafil treatment during 7 days on pharmacologically-evoked baroreflex sensitivity (baroreflex gain; a, *p* < 0.01 vs. sham + vehicle; b, *p* < 0.05 vs. sham + sildenafil; c, *p* < 0.01 vs. 2K1C + vehicle). **(C)** effect of sildenafil treatment during 7 days on spontaneous baroreflex sensitivity (baroreflex gain; a, *p* < 0.01 vs. sham + vehicle; b, *p* < 0.05 vs. sham + sildenafil; c, *p* < 0.05 vs. 2K1C + vehicle).

The representative tracings of changes in blood pressure and heart rate after the administration of vasoactive drugs and baroreflex gain are shown in Figures 2A,B. Vehicle-treated hypertensive animals presented a reduction in baroreflex gain when compared with sham + vehicle rats (−1.93 ± 0.12 vs. −3.63 ± 0.31 bpm/mmHg, *p* < 0.01; Figure 2B). The treatment of hypertensive rats with sildenafil for 7 days was efficient in improving the baroreflex gain (−3.18 ± 0.23 vs. −1.93 ± 0.12 bpm/mmHg, *p* < 0.01). These results were similar in SBRS (Figure 2C). Hypertensive animals presented reduced SBRS when compared with sham + vehicle animals (−1.90 ± 0.22 vs. −3.95 ± 0.46 bpm/mmHg, *p* < 0.01) and the treatment restored SBRS (−3.51 ± 0.29 bpm/mmHg vs. 2K1C + vehicle, *p* < 0.05). In both spontaneous and drug-induced baroreflex activation, treatment of normotensive rats did not modify baroreflex sensitivity (−3.65 ± 0.40 and −3.01 ± 0.33 bpm/mmHg, respectively, *p* > 0.05). Values for SBRS and drug-induced baroreflex gain were not different within groups.

### Treatment with sildenafil for 7 days restores autonomic imbalance in hypertensive rats

**Figure 3:**
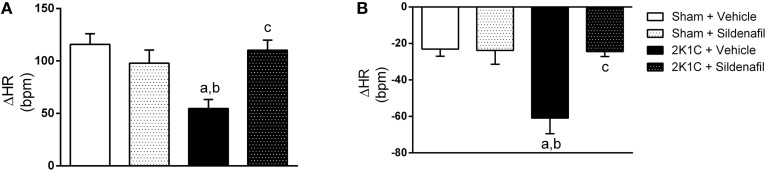
**Effects of treatment with sildenafil on autonomic tonus to the heart**. **(A)** Effect of atropine blockade on resting HR (a, *p* < 0.01 vs. sham + vehicle; b, *p* < 0.05 vs. sham + sildenafil; c, *p* < 0.01 vs. 2K1C + vehicle). **(B)** Effect of propranolol blockade on resting HR (a, *p* < 0.01 vs. sham + vehicle; b, *p* < 0.01 vs. sham + sildenafil; c, *p* < 0.01 vs. 2K1C + vehicle).

To test whether the treatment was efficient in correcting autonomic imbalance in 2K1C animals, sympathetic, and vagal tone to the heart were measured as the ΔHR after pharmacological blockade with propranolol and atropine (Figure 3). Cardiac sympathetic drive was increased in 2K1C + vehicle group when compared with non-treated sham rats (−60.9 ± 8 bpm vs. −23 ± 4 bpm, *p* < 0.01). Moreover, cardiac vagal tone was reduced in hypertension (54.7 ± 8 vs. 115.8 ± 10 bpm, *p* < 0.01). Treatment with sildenafil during 7 days normalized sympathetic (−24.5 ± 3 bpm, *p* < 0.01) and vagal tone (110 ± 9 bpm, *p* < 0.01) in 2K1C hypertension. No differences were found in sham + sildenafil group when compared to sham + vehicle. Changes in MAP after adrenergic blockade with propranolol followed the same pattern as HR (−34.6 ± 2 mmHg in sham + vehicle vs. −60.4 ± 6 mmHg in 2K1C + vehicle, *p* < 0.01 and −36.5 ± 3 mmHg in 2K1C + sildenafil, *p* < 0.01 vs. 2K1C + vehicle). No differences were observed in sham + sildenafil. Regarding muscarinic blockade with atropine, no differences were found in ΔMAP in all groups. In addition, SBRS was evaluated before and after autonomic blockade, as presented in Table 3. No differences were found in SBRS after atropine or propranolol blockade within groups and between different groups.

**Table 3:** **Values of spontaneous baroreflex gain before and after autonomic blockade**.

	**Before blockade**	**After atropine**	**After propranolol**
Sham + Vehicle	−3.95 ± 0.46	−3.52 ± 0.77	−3.73 ± 0.80
Sham + Sildenafil	−3.65 ± 0.40	−3.67 ± 0.63	−4.64 ± 0.42
2K1C + Vehicle	−1.90 ± 0.22^a^, ^b^	−2.41 ± 0.25	−2.73 ± 0.53
2K1C + Sildenafil	−3.51 ± 0.29^c^	−3.62 ± 0.77	−3.23 ± 0.35

*Values were compared by one-way ANOVA for different groups under the same situation and by t-test for the same group before and after blockade*.

a *p < 0.01 vs. sham + vehicle*.

b *p < 0.05 vs. sham + sildenafil*.

c *p < 0.05 vs. 2K1C + vehicle*.

### Sildenafil treatment reduces oxidative stress in hypertensive rats

**Figure 4:**
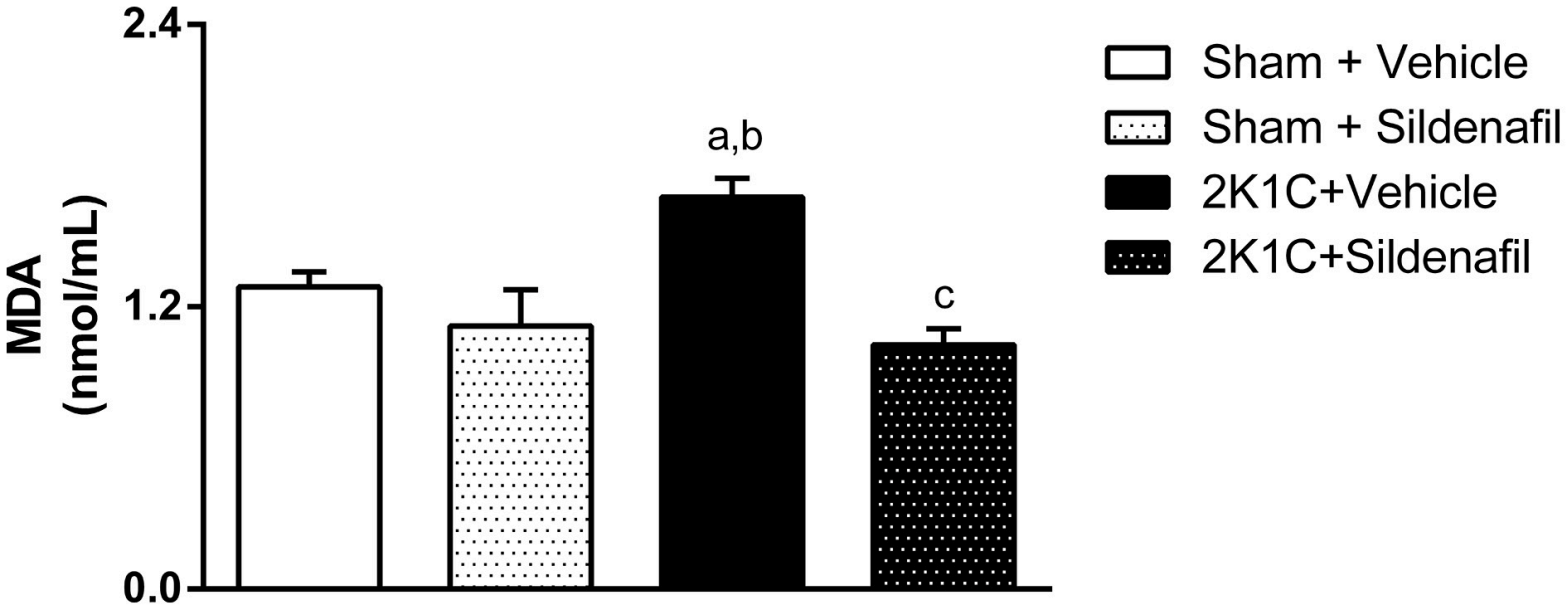
**Sildenafil effects on oxidative stress in hypertensive rats**. Quantification of malondialdehyde in serum of the different groups (a, *p* < 0.05 vs. sham + vehicle; b, *p* < 0.01 vs. sham + sildenafil; c, *p* < 0.01 vs. 2K1C + vehicle).

The quantification of MDA, a final product of lipid peroxidation, is summarized in Figure 4. An increase in MDA concentration of about 30% was observed in non-treated 2K1C animals when compared with controls (1.67 ± 0.08 vs. 1.29 ± 0.06 nmol/mL, *p* < 0.05). Sildenafil treatment given to 2K1C rats for 7 days was able to reduce MDA levels when compared to non-treated hypertensive rats (1.04 ± 0.07 vs. 1.67 ± 0.08 nmol/mL, *p* < 0.05). Treatment of sham animals with sildenafil did not modify this parameter.

## Discussion

In this study we aimed to investigate whether oral treatment with a specific PDE5 inhibitor would improve baroreflex sensitivity in renovascular hypertensive rats. This study is part of a series of studies conducted by our research group, which evaluate the effects of sildenafil administration in animal models of cardiovascular diseases in a translational medicine perspective. Our novel findings are that 7 days of oral sildenafil administration to 2K1C rats were efficient in improving baroreflex control of HR and reestablish autonomic balance to levels of non-hypertensive animals. Furthermore, the treatment decreased serum lipid peroxidation, which is an indicative of oxidative stress. Taken together, these favorable effects of sildenafil treatment were accompanied by a significant reduction in BP in treated hypertensive rats. We propose that sildenafil could be considered as a potential alternative pharmacological approach to treat resistant hypertensive patients.

The efficacy of sildenafil treatment in reducing mean arterial pressure in 2K1C rats is remarkable (Figures 1A,B). This observation is in accordance with previous results by us in 2K1C mice (Dias et al., 2014a,b; Fahning et al., 2015) and by others in rats (Guimarães et al., 2013; Stegbauer et al., 2013). In normotensive rats, sildenafil did not modify blood pressure, which is in accordance with previously described (Ferreira-Melo et al., 2006; Rossoni et al., 2007). In humans, treatment of hypertensive patients with sildenafil during 15 days was able to reduce blood pressure in a degree similar to commonly used antihypertensive drugs, when given as monotherapy (Oliver et al., 2006). It is important to highlight that the reduction in BP elicited by sildenafil treatment was not accompanied by an increase in heart rate (Figure 1C). This was also observed in hypertensive patients that received sildenafil chronically (Oliver et al., 2006).

Previous studies in hypertensive rats found a slight decrease in total peripheral vascular resistance after chronic PDE5 inhibition, with no changes in HR. This suggested that the blood pressure-lowering effect of this drug did not trigger a reflex heart rate response (Ferreira-Melo et al., 2006). This observation is crucial because one concern of sildenafil chronic administration is the potential sympathetic activation, which could limit or blunt BP-lowering effect (Taddei and Ghiadoni, 2006). Although it was described that intracerebroventricular and intrathecal injection of sildenafil acutely increased blood pressure and lumbar sympathetic activity in rats (Fazan et al., 2008; Bombarda et al., 2011) and that, in humans, a single dose of sildenafil increased sympathetically-mediated vascular tone and norepinephrine plasma levels (Dopp et al., 2013), these results are still controversial. Different clinical studies support the idea that orally administrated sildenafil does not increase sympathetic activity in humans (Taddei and Ghiadoni, 2006; Stirban et al., 2009). In the present work we observed that the treatment did not increase sympathetic drive to the heart (Figure 3B). On the contrary, 2K1C + sildenafil animals showed reduced sympathetic drive to the heart when compared with non-treated hypertensive animals.

One important finding from the present work is that sildenafil was efficient in restoring both spontaneous and drug-induced baroreflex sensitivity (Figure 2), which was impaired in 2K1C rats, as previously described (Botelho-Ono et al., 2011; Queiroz et al., 2012). Although the adaptation of Oxford method using single doses of SNP and PHE to elicit drug-induced baroreflex sensitivity may not be the most adequate approach to address baroreflex function, this method is well-accepted in the literature (Braga et al., 2008; Botelho-Ono et al., 2011; Monteiro et al., 2012; Alves et al., 2015). The main limitation of using single bolus doses for vasoactive drugs to evaluate baroreflex function is that it is not possible to build a complete sigmoid curve, which would be more informative. In addition, spontaneous baroreflex was evaluated during different baseline values for each group. This might affect interpretation of the data since each group may be on different parts of the baroreflex curve. However, regardless of the point in the baroreflex curve, our data regarding drug-induced baroreflex sensitivity is supported by the spontaneous baroreflex data, both showing that sildenafil improves baroreflex function in 2K1C rats. It is important to highlight that differences in SBRS were not apparent between groups after autonomic blockade. This can be explained by the fact that, under autonomic blockade, the sequence technique becomes less accurate in estimating baroreflex gain (Stauss et al., 2006).

Nowadays, it is well-accepted that the mechanisms underlying impaired baroreflex control of blood pressure in hypertension involve, at least in part, Ang II-mediated increase in oxidative stress along the axis formed by subfornical organ, paraventricular nucleus of the hypothalamus and rostral ventrolateral medulla (SFO-PVN-RVLM; Braga et al., 2011; de Queiroz et al., 2013). Sildenafil was shown to interfere in renin-angiotensin-aldosterone system (RAAS) during hypertension. Fourteen-days treatment of 2K1C animals with sildenafil resulted in reduction of Ang II in clipped kidney an increasing in Ang 1–7 in kidney and plasma (Dias et al., 2014a,b). Ang 1–7 is a product of the cleavage of Ang II by ACE2. It acts in a G-coupled receptor (Mas receptor) and has opposite effects when compared with Ang II (Santos et al., 2013). Although it was not directly measured in the present work, we cannot rule out the possibility that sildenafil treatment decreases RAAS signaling cascades in brain regions involved in cardiovascular control such as SFO-PVN-RVLM axis.

Interestingly, Aboutabl and colleagues showed that chronic inhibition of PDE5 reduced blood pressure in NO-deficient rats and increased plasma nitrite/nitrate and cGMP levels, suggesting that sildenafil treatment could restore, at least in part, NO/cGMP pathway in this model (Aboutabl et al., 2008). It is important to highlight that oxidative stress and excessive production of reactive oxygen species (ROS) are involved in the decreased bioavailability of NO due to reaction with superoxide anions to form peroxynitrite (Touyz, 2004). It is well-known that ROS and NO play a crucial regulatory role in neurotransmission in important brain areas of cardiovascular control (Kishi et al., 2001; Kishi and Hirooka, 2012; Nishihara et al., 2012a,b). It was previously shown by our group that, in addition to its classic mechanism of action, sildenafil is also capable to increase NO bioavailability in aorta and mesenteric arteriolar cells (Dias et al., 2014a; Leal et al., 2015). If the same occurs in brain, this could explain the amelioration in autonomic imbalance promoted by sildenafil treatment in hypertensive rats (Figure 3). Treated 2K1C animals showed decrease in sympathetic and increase in parasympathetic tone to the heart. The lack of direct measurement of sympathetic nerve activity is a limitation of the study. Whether the amelioration of baroreflex is a cause or a consequence of reduced blood pressure in treated animals is not clear. Also, we cannot rule out the possibility that end organ responses could be different across groups and contribute to responses observed. However, in a translational perspective, the main goal of any antihypertensive therapy is to reduce arterial blood pressure, which was achieved by the treatment in the present work.

In the present study we found that PDE5 inhibition decreased serum lipid peroxidation, a marker of systemic oxidative stress. Increase in serum MDA is associated with increased oxidative stress in RVLM and PVN in 2K1C rats (Campos et al., 2011). The observed antioxidant effect of sildenafil is in accordance with previous investigations by us (Balarini et al., 2013; Rodrigues et al., 2013; Dias et al., 2014b; Fahning et al., 2015) and by others (Koupparis et al., 2005; Shukla et al., 2005; Schäfer et al., 2008; Bivalacqua et al., 2009; Guimarães et al., 2013) in different tissues. We believe that the amelioration of baroreflex sensitivity is directly involved in reducing ROS, since different works from our group (Nunes et al., 2010; Guimarães et al., 2012; Monteiro et al., 2012; Queiroz et al., 2012; Alves et al., 2015) and others (Nishi et al., 2010b, 2013) support the idea that antioxidant treatment with natural or synthetic molecules can enhance baroreflex gain in experimental hypertension. Oxidative stress along SFO-PVN-RVLM pathway is involved in the decreased baroreflex control of blood pressure during hypertension (Braga et al., 2011). Considering that sildenafil can cross the blood-brain barrier and act centrally (Raja and Nayak, 2004), our next step is to investigate whether chronic inhibition of PDE5 with sildenafil centrally decreases oxidative stress in key brain areas involved in cardiovascular control and sympathetic tone. Although it was demonstrated that PDE5 inhibition can decrease oxidative stress due to reduction in the expression of important proteins in the ROS-production cascades (Schäfer et al., 2008), information about similar results in central nervous system is not available yet.

Our findings suggest that right renal artery clipping was efficient in inducing renovascular hypertension (Figure 1). The reduction in right kidney mass was confirmed by the reduction in right kidney weight/body weight ratio in both 2K1C groups. Sildenafil treatment did not modify this parameter (Table 1). Although previous results from our group in 2K1C mice demonstrated that PDE5 chronic inhibition was able to reduce clipped kidney atrophy (Dias et al., 2014a,b), this difference is probably due to the fact that the treatment started 14 days after renal artery clipping, when renovascular hypertension is still developing and is not yet fully established (Navar et al., 1998). Although final body weight was different in sham + sildenafil group, that does not seem to be an effect of the treatment, since it was not observed in 2K1C + sildenafil group nor described in the literature.

In conclusion, this is the first study to show that inhibition of PDE5 is capable to improve baroreflex sensitivity in renovascular hypertensive rats. This improvement is associated with correction of autonomic imbalance in 2K1C rats. It was also documented that sildenafil treatment was efficient in reducing systemic oxidative stress and reducing blood pressure in treated animals. This study reinforces the concept that chronic administration of PDE5 inhibitors could be useful in normalizing blood pressure in resistant patients.

## Author contributions

CC, RA, and AO carried out the experiments, acquisition and analyses of data and drafted the manuscript. JC and MF participated in the supervision of the experiments and critically revised the manuscript. VB participated in the study's design and supervision and in the critical revision of the manuscript. CB conceived the study, supervised and participated in the acquisition and analyses of data and drafted the manuscript. All authors read and approved the final version of the manuscript.

### Conflict of interest statement

The authors declare that the research was conducted in the absence of any commercial or financial relationships that could be construed as a potential conflict of interest.
